# Joint association of indoor allergens, endotoxins, heavy metals, and parabens with allergy-related outcomes in U. S. adults

**DOI:** 10.3389/fpubh.2025.1683381

**Published:** 2025-11-07

**Authors:** Min Xue, Xiaoling Wei, Yun Zhang, Xiang Ma

**Affiliations:** 1 Jinan Institute of Pediatric Research, Children’s Hospital Affiliated to Shandong University (Ji’nan Children’s Hospital), Jinan, Shandong, China; 2 Jinan Key Lab of Respiratory Diseases for Children, Jinan Children’s Hospital, Jinan, Shandong, China; 3 Shandong Provincial Clinical Research Center for Children’s Health and Disease, Jinan Children’s Hospital, Jinan, Shandong, China; 4 Department of Health Data Application and Management, Children’s Hospital Affiliated to Shandong University (Ji’nan Children’s Hospital), Jinan, Shandong, China; 5 Department of Respiratory Medicine, Children’s Hospital Affiliated to Shandong University (Ji’nan Children’s Hospital), Jinan, Shandong, China

**Keywords:** indoor allergens, endotoxin, heavy metals, parabens, allergy-related outcomes, weighted quantile sum (WQS) regression, Bayesian kernel machine regression (BKMR)

## Abstract

**Background:**

While the associations of indoor allergens, endotoxin, heavy metals, and parabens with allergy outcomes are well-studied, the combined association remains unclear. This study examines the association between their combined exposure and allergy outcomes in adults.

**Methods:**

A total of 1,065 adults from the National Health and Nutrition Examination Survey (NHANES) 2005–2006 were included in analyses. We applied a weighted logistic regression model to investigate the association between individual exposure to 10 chemicals (including *Aspergillus fumigatus*, Canf 1, Feld 1, Mus m 1, endotoxin, cadmium, lead, total mercury, methyl paraben, and propyl paraben) and allergy-related outcomes. WQS and BKMR models were further used to examine the combined associations.

**Results:**

The weighted logistic regression model indicated that high-level exposures (Tertile 3 vs. Tertile 1) to Can f 1, Fel d 1, Mus m 1, endotoxin, cadmium, mercury, methyl paraben, and propyl paraben were significantly associated with an increased risk of allergy-related outcomes. In WQS regression analysis, the WQS index was significantly associated with an increased risk of allergy-related outcomes (aOR = 1.49, 95% CI: 1.04–2.11, *p* = 0.027). Can f 1, methyl paraben, endotoxin and cadmium were the most heavily weighed exposure indicators. In BKMR analysis, overall risk estimates were consistently elevated relative to the 25th percentile reference and increased with higher exposure.

**Conclusion:**

Joint and individual exposures to multiple environmental pollutants, particularly Can f 1, methyl paraben, endotoxin, and cadmium, are associated with increased allergy risk in adults. These results underscore the need for integrated exposure assessment in allergy prevention strategies.

## Introduction

Allergy-related diseases include allergic rhinitis (AR), allergic bronchial asthma (referred to as “asthma”), atopic dermatitis (AD), hay fever, and other conditions (1). These diseases affect 40% of the population, and they have been listed by the World Health Organization (WHO) as one of the six most prevalent chronic diseases, making them a key focus of research and prevention in the 21st century (2, 3). Focusing on the research and prevention of allergic diseases is important to improve the quality of life of the affected population.

Allergens, endotoxins, heavy metals, and parabens are ubiquitous environmental pollutants to which humans are commonly exposed. They play important roles in the development and progression of allergy-related diseases. *Aspergillus fumigatus* is a common airborne fungal allergen widely present in indoor environments, including household dust and damp areas (4). Studies have shown that sensitization to *Aspergillus fumigatus* is closely associated with increased asthma severity and airway inflammation (5). Research has also found that nearly all households in the United States, including those without pets, have potential exposure to cat and dog allergens (6). Exposure to cat and dog allergens is strongly associated with sensitization and the development of asthma in children (7, 8). This association is particularly evident in the first 4 years of life, when contact with cats and dogs markedly increases the risk of sensitization (9). In developed countries, the prevalence of sensitization to cat and dog allergens among individuals with symptomatic sensitization may exceed 20% (10). Similarly, detectable levels of the mouse allergen Mus m 1 have been found in approximately 82% of U. S. households (11), and exposure to Mus m 1 has been closely associated with the development of asthma and rhinitis (12). Endotoxins are common components of bioaerosols found in residential, school, and occupational environments. They can remain suspended in the air for extended periods and may resuspend from settled dust due to human activity and environmental changes (13, 14). Endotoxins are typically associated with particulate matter in both indoor and outdoor environments and contribute to the inflammatory effects of these particles (13). They may also trigger or exacerbate asthma and asthma-like symptoms by stimulating the production of pro-inflammatory cytokines (15). Heavy metals such as cadmium, lead, and mercury are common in drinking water, soil, and food chains, especially in meat and seafood (16). Mattila et al. (17) conducted a systematic review that underscored a close relationship between asthma and exposure to cadmium and mercury. Further evidence comes from Yang et al. (18) who reported that higher levels of cadmium and lead in serum were associated with wheezing and reduced lung function in adults. Methyl paraben and propyl paraben are commonly found in cosmetic products, such as shampoo, body lotion, and shower gel. They have also been detected in plastic containers and indoor dust (19, 20). Kim et al. (21) reported that methyl paraben may increase the risk of pruritus in African Americans, while propyl paraben exposure is linked to aeroallergen sensitization (22).

In real-world scenarios, individuals are typically exposed to multiple environmental chemicals simultaneously, rather than to a single pollutant. Allergens and endotoxins often coexist in indoor environments, where they can amplify immune responses and exacerbate allergic diseases (23–25). Similarly, urine or blood samples from the general population frequently contain both heavy metals and parabens. Exposure to heavy metals may exacerbate the risk of allergy-related diseases by inducing oxidative stress and inflammatory responses (26, 27), while parabens, through endocrine disruption, may further alter immune responses (21, 28).

This study systematically analyzes multiple categories of environmental exposures to clarify their cumulative effects and potential synergistic interactions. This framework offers new insights into the mechanisms underlying allergic disease onset and progression and lays a theoretical foundation for more targeted public health prevention strategies.

## Methods

### Study sample

Nation Health and Nutrition Examination Survey (NHANES), a nationally representative program of surveys designed to assess the health and nutritional status of adults and children in US, examined approximately 5,000 non-institutional civilians each year. NHANES was approved by the National Center for Health Statistics (NCHS) Ethics Review Board and had obtained informed consents from participants. The detailed information of NHANES is available at https://www.cdc.gov/nchs/nhanes/index.htm.

**Figure 1:**
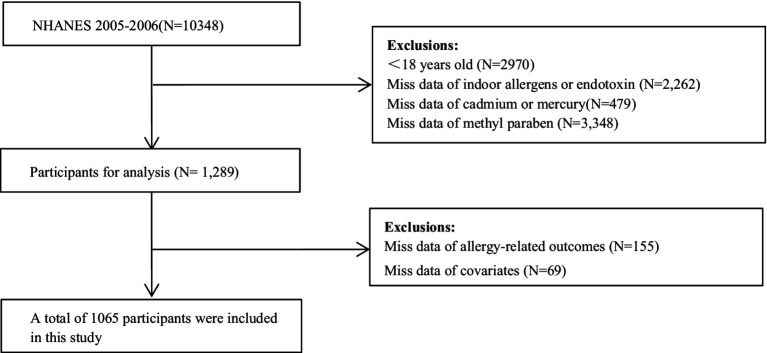
Flowchart of population included in our final analysis (N = 1,065), NHANES, USA, 2005–2006.

We selected 10,348 subjects from NHANES 2005–2006 cycle for eligibility screening. A total of 1,289 adults were left after excluding 2,970 participants aged <18 yeras, 2,262 subjects without data on indoor allergens or endotoxin, 479 subjects without cadmium or total mercury measurements, and 3,348 subjects without. Moreover, 155 subjects without data on allergy-related outcomes and 69 subjects with missing covariates were excluded. Finally, the current cross-sectional study comprised 1,065 adults, and the flow of eligibility screening was shown in Figure 1.

### Measurement of exposure indicators

In this study, we selected exposures measured in the 2005–2006 cycle of the National Health and Nutrition Examination Survey (NHANES) that were potentially relevant to allergic outcomes and environmental exposures. Specifically, we included blood concentrations of heavy metals (cadmium, lead, total mercury); dust concentrations of indoor allergens (Can f 1, Fel d 1, Mus m 1, Bla g 1, Bla g 2, Der p 1, Der f 1, Rat n 1, and Alt a 1), *Aspergillus fumigatus* antigen, and endotoxin; and urinary concentrations of parabens (methyl paraben, butyl paraben, ethyl paraben, and propyl paraben).

Dust samples were collected from participants’ beds and bedroom floors using a Sanitaire Model 3,683 vacuum cleaner equipped with a Mitest Dust Collector and were stored at −80 °C prior to analysis. Eight indoor allergens were quantified using the MARIA® Multiplex Array assay (Indoor Biotechnologies) based on Luminex xMap® technology, while Bla g 1 and *Aspergillus fumigatus* antigen were measured separately by enzyme-linked immunosorbent assays (ELISA) following standard protocols (Indoor Biotechnologies; Greer Laboratories). Dust endotoxin levels were determined using a kinetic chromogenic Limulus amebocyte lysate (LAL) assay conducted at the University of Iowa.

Blood concentrations of cadmium, lead, and total mercury were measured by inductively coupled plasma mass spectrometry (ICP-MS). Urinary parabens were quantified by solid-phase extraction coupled with high-performance liquid chromatography–tandem mass spectrometry (SPE-HPLC-MS/MS), with urinary creatinine (29) measured by a Beckman Synchron CX3 analyzer to adjust for urine dilution.

Values below the limit of detection (LOD) were imputed as LOD divided by the square root of 2 (LOD/√2). Only analytes with detection rates exceeding 70% were included in the final analysis. Detailed laboratory procedures are available in the NHANES Laboratory/Medical Technologists Procedures Manual (30, 31).

### Outcomes and covariation assessment

This study aimed to identify participants with allergy-related outcomes based on two specific criteria: the presence of certain self-reported allergic symptoms and elevated serum total IgE levels. These criteria were selected to capture both localized and systemic allergic responses among the study population.

In the NHANES 2005–2006 survey, participants were asked about a range of common allergic conditions, including wheezing, asthma, hay fever, general allergies, rhinitis, itchy rash, and eczema. Participants who reported any of these symptoms /diseases, regardless of their IgE levels, were classified as having allergy-related outcomes. Participants without self-reported symptoms but with elevated serum total IgE (≥150 kU/L) were likewise classified as having allergy-related outcomes. A serum total IgE ≥ 150 kU/L was considered indicative of an allergic condition (3).

Covariates, including gender, age, body mass index (BMI), race, educational level, annual household income, and drinking and smoking status, were collected via interview by NHANES. BMI was calculated as weight in kilograms divided by the square of height in meters (kg/m^2^). Age, BMI and serum cotinine were analyzed as continuous variables. The remaining covariates were categorized as follows: sex (female and male), race (Mexican American, non-Hispanic black, non-Hispanic white, and Other Race), educational level (below high school, high school, and above high school), annual household income (<$20,000 and ≥$20,000), and alcohol (drinker and non-drinker). Urinary creatinine was used to normalize analyte concentrations and adjust for dilution.

### Statistical analysis

In this study, continuous variables were expressed as weighted mean ± SE, while categorical variables were presented as percentages and counts. Baseline characteristics were compared using *t*-tests for continuous variables and *χ*^2^ tests for categorical variables. Ten exposure indicators were log-transformed to alleviate the skewed distribution, and Spearman correlation was used to examine correlations among them. Multivariable logistic regression models was applied to examine the associations between adult exposure to ten specific chemicals and allergic diseases. These chemicals were log-transformed and divided into tertiles (Q1–Q3) for analysis.

The findings are expressed as odds ratios (ORs) with their respective 95% confidence intervals (CIs). A three-node restricted cubic spline (RCS) logistic regression model was used to study the potential dose–response relationships between ten chemicals and allergic diseases, using the median as the reference point.

In environmental epidemiology, the WQS regression model is widely applied to assess the effects of multiple exposures on specific health outcomes. The statistical model creates a weighted index for correlated chemical mixtures by using the chemical components’ quantiles. It assesses the link between the outcome and the index, estimating the mixture exposure’s overall effects and each predictor’s contributions to the index effect (32). The present study employed WQS regression to investigate the correlation between allergic diseases and mixtures of ten chemicals. The WQS index was constructed on the basis of the quartiles of the levels of the ten chemicals, allocating 40% of the sample as a training set and the remaining 60% as the validation set. Since no initial assumptions were made about the direction of the relationships between the levels of ten chemicals and allergic diseases, WQS regressions were conducted, allowing all components of the index to contribute either positively or negatively. These analyses adjusted for all previously described covariates. To enhance the sensitivity in identifying significant predictors and to achieve stable weights, 100 bootstrap samples were utilized. A threshold of 1/10 = 0.1 was established for weights to identify the relatively important congeners affecting the overall mixture effect, with 10 denoting the number of indicators (32). The interpretation of the log-transformed WQS index suggested an escalation in the risk of allergic diseases corresponding to each increase in quartile of the chemical mixture level.

Mixture modeling analysis based on Bayesian Kernel Machine Regression (BKMR) was conducted to assess the joint effects of multiple chemical exposures on allergy-related outcomes, as well as to explore potential nonlinear dose–response relationships and interactions among exposures (33). Given the correlations among the exposures, hierarchical variable selection was performed using a Markov chain Monte Carlo algorithm with 10,000 iterations to estimate posterior inclusion probabilities (PIPs), including both group-level PIPs (groupPIP) and conditional-level PIPs (condPIP). A PIP threshold of 0.5 was used to identify important contributors within the mixture. Based on Pearson’s correlation coefficients and similarities in exposure sources, the chemicals were categorized into three groups: group 1 included indoor allergens (Can f 1, Fel d 1, and Mus m 1), *Aspergillus fumigatus*, and endotoxin; group 2 included heavy metals (cadmium, lead, and total mercury); and group 3 included parabens (propyl paraben and methyl paraben). For the estimation of the overall mixture effect, the 25th percentile of all exposures was set as the reference level, and overall risk estimates were evaluated across increasing percentiles in 5-percentile increments. Single-exposure response functions were assessed by varying each chemical at the 25th, 50th, and 75th percentiles while holding others at their median levels. Bivariate exposure-response functions were further used to explore potential interactions between chemical pairs. All statistical analyses were performed using R software (version 4.0.2; R Foundation for Statistical Computing, Vienna, Austria). Multivariable logistic regression, restricted cubic spline (RCS) modeling, weighted quantile sum (WQS) regression, and BKMR modeling were conducted using the “rms” (version 6.7.1), “gWQS” (version 3.0.5), and “bkmr” (version 0.2.2) packages. A two-sided *p*-value < 0.05 was considered statistically significant.

## Results

### Population characteristics and exposure indicators distribution

The study included 1,065 participants, of whom 705 (66.2%) had allergy-related outcomes. Table 1 summarizes participants’ baseline characteristics. The weighted mean age was 44.92 years, and 535 (50.23%) were male. Allergic cases were more likely to be non-Hispanic white, well-educated, from higher household income groups, and current nondrinkers, and they had significantly higher BMI and serum cotinine levels compared with non-cases. Based on the data presented in Supplementary Table S1, among the indoor allergens and endotoxin measured in dust samples and heavy metals detected in blood, *Aspergillus fumigatus* had the highest concentration at 123.842 μg/g dust, followed by endotoxin at 15.612 EU/mg dust. Lead had higher levels than cadmium and total mercury, with concentrations of 14.100 μg/L, 0.320 μg/L, and 0.870 μg/L, respectively. In terms of parabens in urine, methyl paraben showed the highest concentration at 81.176 μg/g, followed by propyl paraben at 11.275 μg/g. Supplementary Figure S1 shows the correlations among log-transformed indoor allergens, endotoxin, heavy metals, and parabens. Methyl and propyl parabens were strongly correlated (r = 0.808, *p <* 0.001). Can f 1 was moderately correlated with Fel d 1 (r = 0.371, *p <* 0.001). Lead was also significantly correlated with cadmium (r = 0.350, *p <* 0.001). These findings suggest potential co-exposure patterns among environmental pollutants.

**Table 1:** Demographic characteristics of U. S. adults by allergic and non-allergic status, NHANES 2005–2006.

Characteristics^a^	Participants			*p*-value
	Allergic group = 1 (*n* = 705)	Non-allergic group = 0 (*n* = 360)	Total (*n* = 1,065)	
Gender				
Male	358 (50.78)	177 (49.17)	535 (50.23)	0.665
Female	347 (49.22)	183 (50.83)	530 (49.77)	
Age (y), weighted mean (SE)	45.64 (19.75)	43.50 (19.52)	44.92 (19.69)	0.094
BMI (kg/m^2^), weighted mean (SE)	29.35 (8.17)	27.77 (6.27)	28.82 (7.62)	0.001
Race				
Mexican American	128 (18.16)	92 (25.56)	220 (20.66)	0.045
Non-Hispanic Black	183 (25.96)	86 (23.89)	269 (25.26)	
Non-Hispanic White	345 (48.94)	158 (43.89)	503 (47.23)	
Other Race	49 (6.95)	24 (6.67)	73 (6.85)	
Education level				
Below than high school	184 (26.10)	130 (36.11)	314 (29.48)	0.001
High school	191 (27.09)	96 (26.67)	287 (26.95)	
Above than high school	330 (46.81)	134 (37.22)	464 (43.57)	
Annual household income ($)				
<20,000	203 (28.79)	80 (22.22)	283 (26.57)	0.026
≥20,000	502 (71.21)	280 (77.78)	782 (73.43)	
Alcohol				
Drinker	451 (63.97)	201 (55.83)	652 (61.22)	0.012
Non-drinker	254 (36.03)	159 (44.17)	413 (38.78)	
Serum cotinine (ng/mL), weighted mean (SE)	71.38 (142.61)	53.93 (116.79)	65.50 (134.67)	0.046

^a^Normally distributed continuous variables were expressed as mean ± SD; Categorical variables were presented as n (%). *n*, numbers of subjects; %, weighted percentage.

### Single allergens, endotoxin, heavy metals, and parabens exposure with allergy-related outcomes

The highest tertile (T3) levels of Can f 1, Fel d 1, Mus m 1, endotoxin, cadmium, total mercury, methyl paraben, and propyl paraben were associated with elevated risk of allergy-related outcomes compared to the lowest tertile (T1) levels [Can f 1: (OR: 1.37, 95% CI: 1.27–1.47), Fel d 1: (OR: 1.15, 95% CI: 1.07–1.23), Mus m 1:(OR: 1.29, 95% CI: 1.20–1.37), endotoxin: (OR: 1.21, 95% CI: 1.13–1.30), cadmium: (OR: 1.87, 95% CI: 1.73–2.03), total mercury: (OR: 1.14, 95% CI: 1.06–1.22), methyl paraben: (OR: 1.49, 95% CI: 1.35–1.65), propyl paraben: (OR: 1.16, 95% CI: 1.05–1.28)] (Table 2).

**Table 2:** Association of allergens, endotoxin, heavy metals, parabens with allergy-related outcomes, NHANES, 2005–2006.

Variables^a^	Tertile 1	Tertile 2		Tertile 3	
		OR (95% CI)	*p*-value	OR (95% CI)	*p*-value
*Aspergillus fumigatus* (μg/g dust)	Reference	1.08 (1.02–1.15)	0.015	1.08 (1.02–1.15)	0.015
Can f 1 (μg/g dust)	Reference	1.75 (1.63–1.88)	<0.001	1.75 (1.63–1.88)	<0.001
Fel d 1 (μg/g dust)	Reference	1.03 (0.96–1.11)	0.385	1.03 (0.96–1.11)	0.385
Mus m 1 (μg/g dust)	Reference	1.18 (1.11–1.25)	<0.001	1.18 (1.11–1.25)	<0.001
Endotoxin (EU/mg dust)	Reference	1.11 (1.04–1.18)	0.002	1.11 (1.04–1.18)	0.002
Cadmium (μg/L)	Reference	1.13 (1.06–1.21)	<0.001	1.13 (1.06–1.21)	<0.001
Lead (μg/dL)	Reference	1.08 (1.01–1.16)	0.033	1.08 (1.01–1.16)	0.033
Total Mercury (μg/L)	Reference	1.28 (1.2–1.37)	<0.001	1.28 (1.2–1.37)	<0.001
Methyl paraben (μg/g)	Reference	0.85 (0.79–0.92)	<0.001	0.85 (0.79–0.92)	<0.001
Propyl paraben (μg/g)	Reference	1.39 (1.28–1.51)	<0.001	1.39 (1.28–1.51)	<0.001

^a^Model was adjusted for sex, age, BMI, race, marital status, education level, annual household income, alcohol and serum cotinine, The 10 exposure indicators metabolites have undergone log transformation.

RCS regression was employed to visualize the log-linear dose–response relationship between levels of allergens, endotoxin, heavy metals, environmental parabens and risk of allergy-related outcomes. The results, illustrated in Supplementary Figure S2, showed statistically significant linear associations between allergy risk and the concentrations of *Aspergillus fumigatus*, Mus m 1, endotoxin, cadmium, lead, total mercury, methyl paraben, and propyl paraben. Additionally, the risks associated with Can f 1 and Fel d 1 demonstrated notable nonlinear correlations (*p* for nonlinearity < 0.05).

### Allergens, endotoxin, heavy metals, parabens co-exposure and allergy-related outcomes

**Figure 2:**
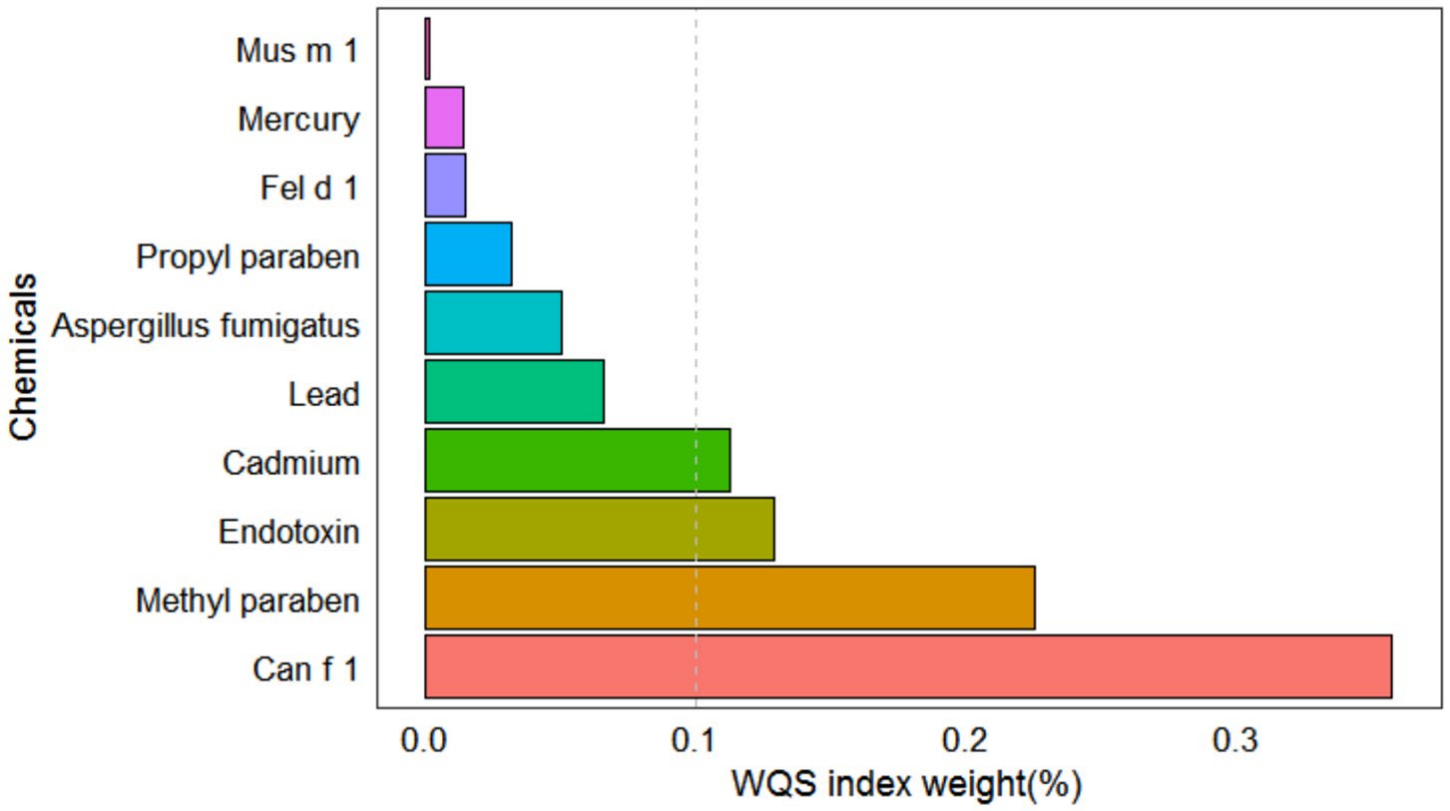
WQS model regression index weights for allergy-related outcomes. Models were adjusted for sex, age, BMI, race, education level, annual household income, alcohol and serum cotinine, and log-transformed creatinine.

**Figure 3:**
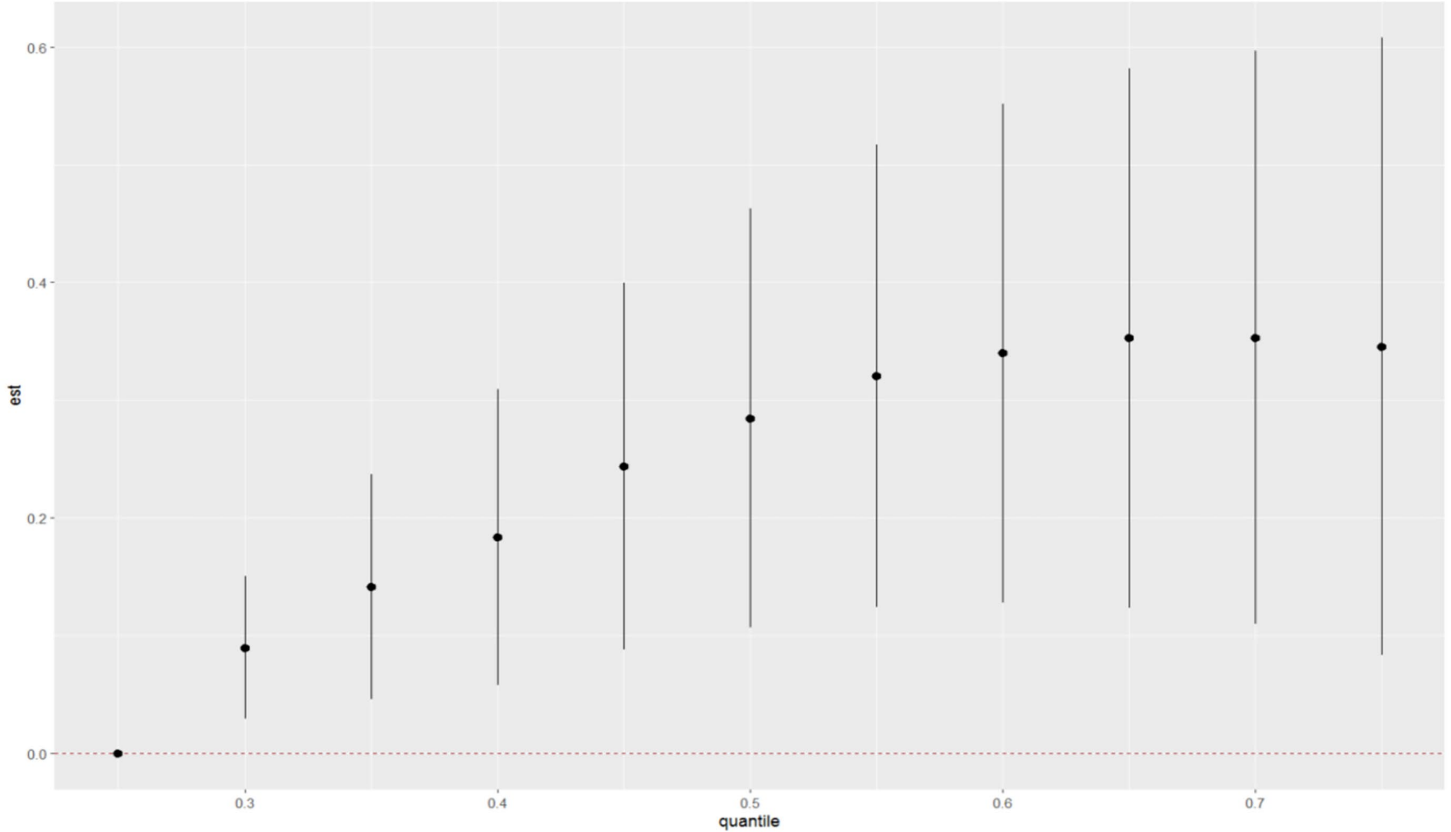
The joint effects of allergens, endotoxin, heavy metals and parabens on allergy-related outcomes risk were estimated by BKMR models in total population.

After adjusting for all covariates, the WQS model showed a statistically significant positive association between co-exposure to ten chemicals and allergy-related outcomes (OR: 1.49, 95% CI: 1.04–2.11, Figure 2). Among the individual exposure indicators, Can f 1 contributed mostly to the association (Table 3). No significant negative association was observed between the exposure mixture and allergy-related outcomes (the results are not shown). The ten exposures were categorized into three groups according to their source, and the BKMR model was applied to investigate the relationship between simultaneous exposures to these indicators and allergy-related outcomes. The posterior inclusion probabilities (PIPs) derived from the BKMR model for the three groups (groupPIP) and each exposure indicators (condPIP) are summarized in Table 4. The PIPs for the allergen, heavy-metal, and paraben groups were 0.973, 0.438, and 0.349, respectively. Within the allergen group, Can f 1 had the strongest contribution (CondPIP = 0.934). In the heavy-metal group, lead drove the main effect of the whole group (CondPIP = 0.462), and propyl paraben drove the main effect in the environmental parabens group (CondPIP = 0.611). Figure 3 illustrates the overall relationship between the mixtures of exposure indicators and the heightened risk of allergy-related outcomes as analyzed using the BKMR model. The analysis showed that allergy-related risk increased significantly with higher concentrations of allergens, endotoxin, heavy metals, and parabens.

**Table 3:** The estimated weights of eposure indicators in WQS models.

Variables	Weight
Can f 1 (μg/g dust)	0.357
Methyl paraben (μg/g)	0.225
Endotoxin (EU/mg dust)	0.129
Cadmium (μg/L)	0.112
Lead (μg/dL)	0.066
*Aspergillus fumigatus* (μg/g dust)	0.050
Propyl paraben (μg/g)	0.032
Fel d 1 (μg/g dust)	0.015
Total Mercury (μg/L)	0.014
Mus m 1 (μg/g dust)	0.001

**Table 4:** Bayesian Kernel machine regression (BKMR) model of posterior inclusion probabilities (PIPs) for groups (group PIPs), and (condPIPs) for each exposure indicator metabolites.

Variable^a^	Group	groupPIP	condPIP
*Aspergillus fumigatus* (μg/g dust)	1	0.973	0.004
Can f 1(μg/g dust)	1	0.973	0.934
Fel d 1(μg/g dust)	1	0.973	0.026
Mus m 1(μg/g dust)	1	0.973	0.004
Endotoxin (EU/mg dust)	1	0.973	0.032
Cadmium (μg/L)	2	0.438	0.328
Lead (ug/dL)	2	0.438	0.462
Total Mercury (μg/L)	2	0.438	0.211
Propyl paraben (μg/g)	3	0.349	0.611
Methyl paraben (μg/g)	3	0.349	0.389

^a^Model was adjusted for sex, age, BMI, race, education level, annual household income, alcohol and serum cotinine, The 10 exposure indicators metabolites have undergone log transformation.

The univariate exposure–response functions of the ten chemicals were plotted, showing similar effect directions to those observed in the single-analyte GLMs (Supplementary Figure S3). To further explore potential interactions, pairwise cross-sections of the exposure–response functions were generated (Supplementary Figure S4). While two curves showed similar trends and one curve deviated, the overall evidence did not support the presence of a strong interaction.

## Discussion

This study found that among various environmental exposures, Can f 1, methyl paraben, and endotoxin were most strongly associated with allergy-related outcomes. Multi-pollutant analyses further supported their relevance under combined exposure conditions, with Can f 1 contributing the most to the overall risk profile. These findings highlight that, in real-world co-exposure contexts, multiple environmental factors may jointly influence the development of allergic diseases, underscoring the need for an integrated assessment of health risks from multiple exposure sources.

Our study focused on common indoor allergens—Can f 1, Fel d 1, Mus m 1, and *Aspergillus fumigatus*—which are widely detected in household dust and indoor air (4, 6, 11). We also examined endotoxins, which, while not allergens themselves, can still exacerbate respiratory outcomes by activating innate immune pathways (34, 35). Our results showed that the median concentrations of Can f 1, Fel d 1, and Mus m 1 were 0.164, 0.099, and 0.014 μg/g dust, respectively. These values were significantly lower than those reported in French mattress dust (2.9 and 4.3 μg/g) (36), in mouse allergens from Strasbourg, France (10 ng/g) (37), and in living room floor dust from 216 households in New Zealand (11 ng/g) (38). The endotoxin concentration in this study was 15.612 EU/mg, which is lower than the concentrations reported in Denmark and Palestine, where levels in mattress and floor dust were 25.7 EU/mg and 49 EU/mg, respectively (39, 40). These findings suggest that allergen and endotoxin concentrations are generally low in U. S. households. Despite relatively low concentrations, Can f 1 was significantly associated with an increased risk of allergy-related outcomes. Endotoxin exposure was also positively associated with risk, and Fel d 1 showed similar results, consistent with previous studies. Numerous studies have shown that exposure to dog and cat allergens is significantly linked to a heightened risk of allergy-related outcomes in adults, such as asthma, rhinitis, and atopic dermatitis (41–43). NHANES-based studies further confirmed that Can f 1 are associated with an increased incidence of allergic rhinitis, whereas Fel d 1 and endotoxins are related to asthma severity and wheezing (15, 24, 44). Although *Aspergillus fumigatus* and Mus m 1 have shown a certain degree of positive correlation in allergic diseases, consistent with previous studies (45, 46), their effects appear attenuated in mixed exposure environments. This attenuation may be attributed to adaptive immune responses or differing mechanisms of action among the co-exposed allergens, leading to a diminished overall effect in the context of combined exposures.

In addition, heavy metals play an important role in the development of allergy-related diseases. In our study, the geometric mean blood cadmium concentration was 0.366 μg/L, slightly higher than that reported in the general Canadian population (0.35 μg/L) (16), but lower than in Germany (0.58 μg/L) (47) and the non-occupational population of Quebec City (0.69 μg/L) (48). Although exposure levels were generally in the low to moderate range, we still observed a significant association between cadmium exposure and allergy-related outcomes, suggesting that even moderate environmental exposure may have adverse effects on allergic health. This finding is consistent with previous studies. Cadmium exposure has been linked to symptoms such as itchy eyes and coughing in patients with chronic cough, possibly through non-IgE-mediated mechanisms (49). In addition, cadmium has been associated with the onset and exacerbation of asthma and allergic rhinitis (26, 50, 51), and studies in U. S. populations have found that cadmium increases the risk of wheezing and asthma among current smokers, and is associated with reduced lung function in nonsmokers (18). In this study, the geometric mean blood lead concentration among adults was 14.026 μg/L. Compared with reports from other countries, this level was notably higher than that of German adults (10 μg/L) (52), lower than that reported in the Chinese population (20.66 μg/L) (53), and approximately equivalent to the level observed in the Japanese population (around 14.0 μg/L). These regional differences may reflect variations in environmental pollution, occupational exposure frequency, dietary patterns, and national regulatory policies. Regarding the association with allergy-related outcomes, lead exposure showed no significant relationship in the single-exposure model, and the RCS analysis indicated a slight but statistically non-significant negative trend at higher concentrations. In mixture analyses, the BKMR model assigned a moderate conditional posterior inclusion probability (condPIP) to lead; however, inconsistent findings across models suggest that lead may have a limited overall effect on allergy-related outcomes. These results are generally consistent with previous research. For example, Yang et al. (18) also found no significant association between lead exposure and allergic diseases. Although some epidemiological and occupational studies have suggested that lead may interfere with immune function by altering immunoglobulin levels or promoting inflammatory cytokine expression (54), its associations with asthma, allergic rhinitis, and eczema remain inconclusive. It is important to note that the immunological effects of lead may be influenced by multiple factors, including exposure dose, physiological status, and genetic background (55, 56). Therefore, while this study did not identify lead as a major contributor to allergy risk, its potential immunotoxic effects under specific exposure conditions or among susceptible populations warrant further investigation.

Meanwhile, the present study found that the geometric mean blood mercury concentration in the U. S. population was 0.894 μg/L. This level was higher than that reported in Germany (0.58 μg/L) (47) and in a non-occupationally exposed population from the Quebec City region (0.74 μg/L) (48), but slightly lower than the Canadian national average (0.91 μg/L) (16). These regional differences likely reflect the influence of environmental contamination, dietary habits (e.g., seafood and rice intake), and lifestyle factors. In the U. S., about 90% of total mercury intake originates from the consumption of shellfish and sea fish, and rice also serves as an important source of methylmercury exposure (57, 58). Existing studies suggest that mercury exposure may be associated with contact dermatitis, allergic rashes, and occupational allergic contact dermatitis (59–61), and may also induce inflammatory and autoimmune responses (62). However, other studies have found no significant association with asthma, allergic rhinitis, or atopic dermatitis (26). Our study further found that the risk of allergy-related outcomes increased significantly at low to moderate levels of mercury exposure, but plateaued or slightly declined at higher concentrations. This pattern aligns with previous experimental evidence showing that methylmercury (MeHg) can enhance the expression of inflammatory cytokines at low to moderate doses, while exerting notable immunosuppressive effects at higher doses (63). Wild et al. (64) similarly reported that low-dose MeHg exposure during gestation and lactation enhanced immune activity in offspring, whereas high-dose exposure led to immune suppression. Although mercury contributed relatively less within the overall mixture of environmental pollutants, its potential for chronic immune disruption should not be overlooked, particularly among highly exposed or immunologically vulnerable populations.

This study found that both methyl paraben and propyl paraben were associated with an increased risk of allergy-related outcomes. Methyl paraben consistently showed stable and significant positive associations across multiple models, suggesting a potential independent sensitizing effect. In contrast, propyl paraben exhibited a weaker association in single-exposure models but contributed more substantially in mixture models, possibly reflecting a cumulative effect in the context of co-exposure to multiple environmental chemicals. Regarding exposure levels, the geometric means of methyl and propyl parabens in this population were 64.46 μg/g and 8.95 μg/g, respectively. These values are comparable to previous reports from U. S. populations (65), but notably higher than those observed in Chinese (16.3–18.4 and 0.93–1.62 μg/g, respectively) (66) and Japanese populations (37.4 and 0.89 μg/g, respectively) (67). Such differences may reflect regional variation in personal care product use, lifestyle, and regulatory policies. Our findings are consistent with previous epidemiological studies. For example, an analysis based on 2005–2006 NHANES data reported a significant association between methyl paraben exposure and increased risk of itchy rash among African American individuals (21). Another study found that propyl paraben exposure was significantly associated with increased sensitivity to airborne allergens and positively correlated with eczema area and severity index (EASI) scores (22), suggesting a potential role in the development and progression of allergic diseases.

We did not observe statistically significant interactions between the exposures examined. Nonetheless, the biological convergence of effects—endocrine/immune dysregulation attributable to parabens, TLR4–NF-κB–mediated innate inflammatory activation by endotoxin, oxidative stress and epithelial-barrier injury induced by heavy metals, and Th2-driven sensitization elicited by indoor allergens—provides a plausible rationale for potential interaction effects under real-world co-exposures. Meanwhile, allergic disease may also be influenced by protective exposures. Evidence indicates that certain diet- and environment-derived metabolites—such as omega-3 fatty acids, microbial tryptophan-derived indole derivatives, and various flavonoids—can attenuate allergic inflammation by modulating adaptive and innate immune pathways and maintaining epithelial-barrier integrity (68–70). As these exposures were not included in the present study, future work should jointly model risk and protective exposures within a mixture framework to estimate net effects and refine public health recommendations..

This study has notable strengths. It is based on data from a nationally representative sample of U. S. adults, enhancing the generalizability of the findings. To our knowledge, this is the first study to comprehensively assess the associations between combined exposures to multiple common environmental factors—including indoor allergens, endotoxins, heavy metals, and parabens—and allergy-related outcomes. These findings provide important evidence for understanding environmental determinants of allergic diseases and provide a foundation for future longitudinal and mechanistic investigations.

Several limitations of this study should be acknowledged. First, the cross-sectional design limits the ability to infer causal relationships between environmental exposures and allergy-related outcomes. Second, although multiple covariates were adjusted for, the potential for residual confounding cannot be ruled out. Third, environmental exposures were measured using different biological samples, with blood reflecting longer-term exposure and urine reflecting more recent exposure. Moreover, all biomarkers were based on a single measurement, which may not fully represent long-term exposure levels. Finally, this study relies on data from the NHANES 2005–2006 cycle. Exposure patterns to chemicals such as parabens and lead may have evolved since data collection, potentially limiting the generalizability of our findings to the contemporary U. S. population..

In conclusion, this study underscores the complex links between environmental exposures and allergy outcomes in adults. Can f 1, Methyl paraben, endotoxin, and cadmium were linked to higher allergy risks. Continued surveillance of these effects is vital for guiding targeted interventions and public health policies.

## Data Availability

Publicly available datasets were analyzed in this study. This data can be found at: https://www.cdc.gov/nchs/nhanes/index.htm.
