# Correction: Joint association of indoor allergens, endotoxins, heavy metals, and parabens with allergy-related outcomes in U. S. adults

**DOI:** 10.3389/fpubh.2025.1761697

**Published:** 2026-01-06

**Authors:** Frontiers Production Office

**Affiliations:** Frontiers Media SA, Lausanne, Switzerland

**Keywords:** indoor allergens, endotoxin, heavy metals, parabens, allergy-related outcomes, weighted quantile sum (WQS) regression, Bayesian kernel machine regression (BKMR)

There was a mistake in Figure 1 as published. In the Exclusions section of Figure 1, “Miss data of urinary triclosan or methyl paraben (*N* = 3,348)” has been revised to “Miss data of methyl paraben (*N* = 3,348)”.

The corrected Figure 1 appears below.

**Figure 1:**
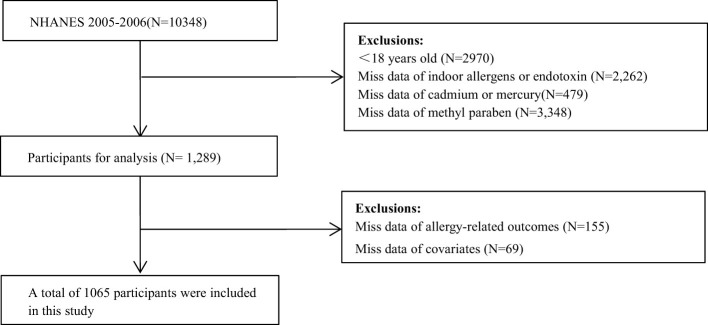
Flowchart of population included in our final analysis (*N* = 1,065), NHANES, USA, 2005–2006.

There was a mistake in the captions of Figures 2 and 3 as published. The figure legends were in the wrong order. The corrected captions for Figures 2 and 3 appear below.

Figure 2. WQS model regression index weights for allergy-related outcomes. Models were adjusted for sex, age, BMI, race, education level, annual household income, alcohol and serum cotinine, and log-transformed creatinine.

Figure 3. The joint effects of allergens, endotoxin, heavy metals and parabens on allergy-related outcomes risk were estimated by BKMR models in total population.

There was a mistake in the publisher location and publisher name for references [30, 31, and 48] as published. The corrected publisher location and publisher name for references [30, 31, and 48] appears below.

Ref. 30: *Laboratory Procedure Manual (Method No: 6301.01)*. Atlanta, GA: Centers for Disease Control and Prevention (CDC) (2021).

Ref. 31: *Laboratory Procedure Manual (Method No: 6306.03)*. Atlanta, GA: Centers for Disease Control and Prevention (CDC) (2012).

Ref. 48: *Étude sur l’établissement de valeurs de référence d’éléments traces et de métaux dans le sang, le sérum et l’urine de la population de la grande région de Québec*. Québec, QC: Institut National de Santé Publique du Québec (2003).

The original version of this article has been updated.

## Generative AI statement

Any alternative text (alt text) provided alongside figures in this article has been generated by Frontiers with the support of artificial intelligence and reasonable efforts have been made to ensure accuracy, including review by the authors wherever possible. If you identify any issues, please contact us.

