# Supplementary material for: Joint association of indoor allergens, endotoxins, heavy metals, and parabens with allergy-related outcomes in U. S. adults
Source: Front Public Health. 2025 Nov 7;13:1683381. doi: 10.3389/fpubh.2025.1683381 (PMC12634600; doi:10.3389/fpubh.2025.1683381)
Supplement: Supplementary file 1 [file Data_Sheet_1.pdf]

Supplementary Material

1.Supplementary Tables

Supplementary Table S1. Distribution of the Exposure Indicators in NHANES 2005–2006 (N = 1065).

Exposures[a]	DF(%)	GM	Mean	Min	P33	P50	P67	Max
Aspergillus fumigatus (µg/g dust)	74.08	108.193	150.905	12.624	68.692	123.842	168.737	1690.925
Can f 1 (µg/g dust)	78.70	0.298	12.413	0.009	0.04	0.164	1.077	596.221
Fel d 1 (µg/g dust)	88.89	0.212	24.563	0.003	0.037	0.099	0.424	1530.137
Bla g 1 (U/g dust)	34.84	0.481	1.454	0.222	0.222	0.222	0.444	109.545
Bla g 2 (µg/g dust)	27.79	0.193	0.217	0.154	0.154	0.154	0.154	1.830
Der p 1 (µg/g dust)	49.67	0.066	1.11	0.009	0.009	0.026	0.175	52.05
Der f 1 (µg/g dust)	56.53	0.049	1.153	0.009	0.009	0.009	0.074	126.082
Mus m 1 (µg/g dust)	80.19	0.017	0.230	0.002	0.005	0.014	0.034	21.718
Rat n 1 (µg/g dust)	25.51	0.004	0.011	0.003	0.003	0.003	0.003	2.739
Alt a 1 (µg/g dust)	20.85	0.004	0.005	0.003	0.003	0.003	0.003	0.257
Endotoxin (EU/mg dust)	99.82	14.469	37.428	0.0003	10.144	15.612	25.881	3205.045
Cadmium (µg/L)	100	0.366	0.541	0.140	0.231	0.320	0.470	8.550
Lead (ug/L)	99.25	14.026	18.036	1.800	9.912	14.100	19.800	122.000
Mercury (µg/L)	100	0.894	1.396	0.140	0.600	0.870	1.300	26.000
Methyl paraben (µg/g)	98.87	64.461	220.596	0.376	26.532	81.176	179.671	5285.714
Butyl paraben (µg/g)	48.26	0.458	4.496	0.037	0.132	0.243	0.618	165.969
Ethyl paraben (µg/g)	43.94	0.019	0.139	0.001	0.007	0.011	0.026	8.675
Propyl paraben (µg/g)	93.24	8.946	60.236	0.043	2.442	11.275	37.913	4423.313

"DF Detection frequency, GM Geometric mean; Models were adjusted forsex, age, BMI, race, education level, annual household income, alcohol and serum cotinine. Continuous, ln-transformed concentration of variables.

2. Supplementary Figures

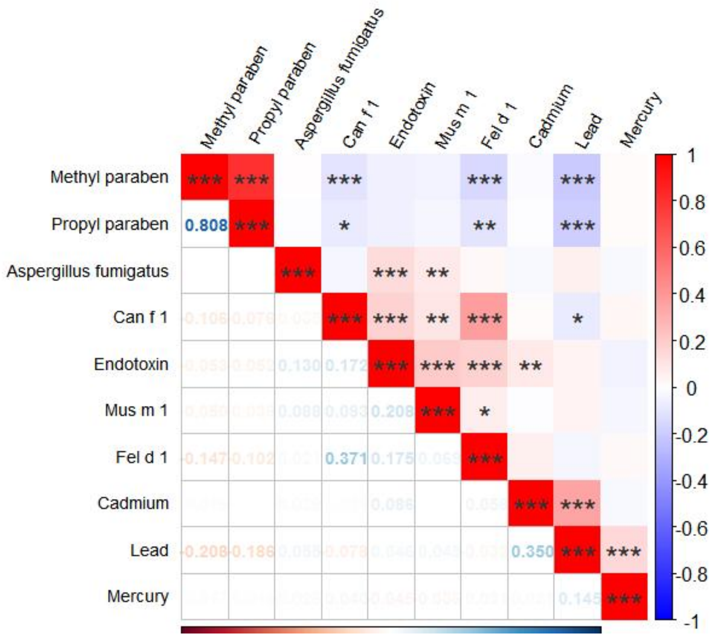

Supplementary Figure S1 Mutual correlations among the 10 exposure indicators . The numbers represented the correlation coefficients, and blue and red represented positive and negative correlations, respectively

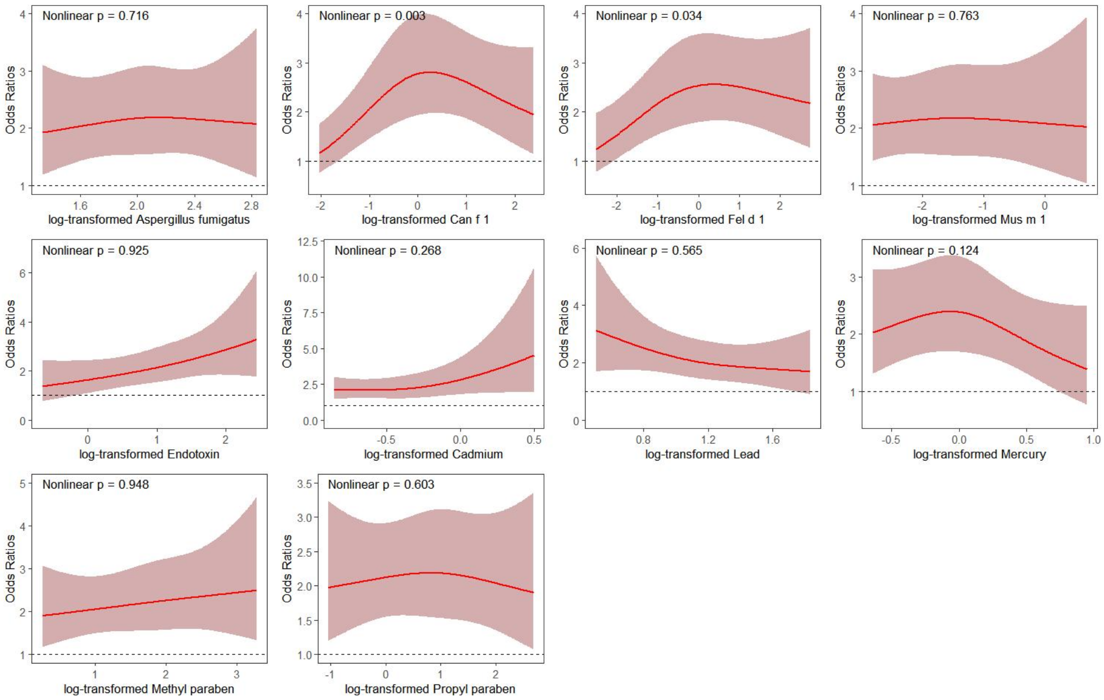

Supplementary Figure S2 Restricted cubic spline (RCS) plot of the association between log-transformed exposure indicators metabolites levels and allergy-related outcomes. RCS regression was adjusted for sex, age, BMI, race, education level, annual household income, alcohol and serum cotinine. The solid line and the shadow around it represent the odds ratios and 95% confdence intervals, respectively.

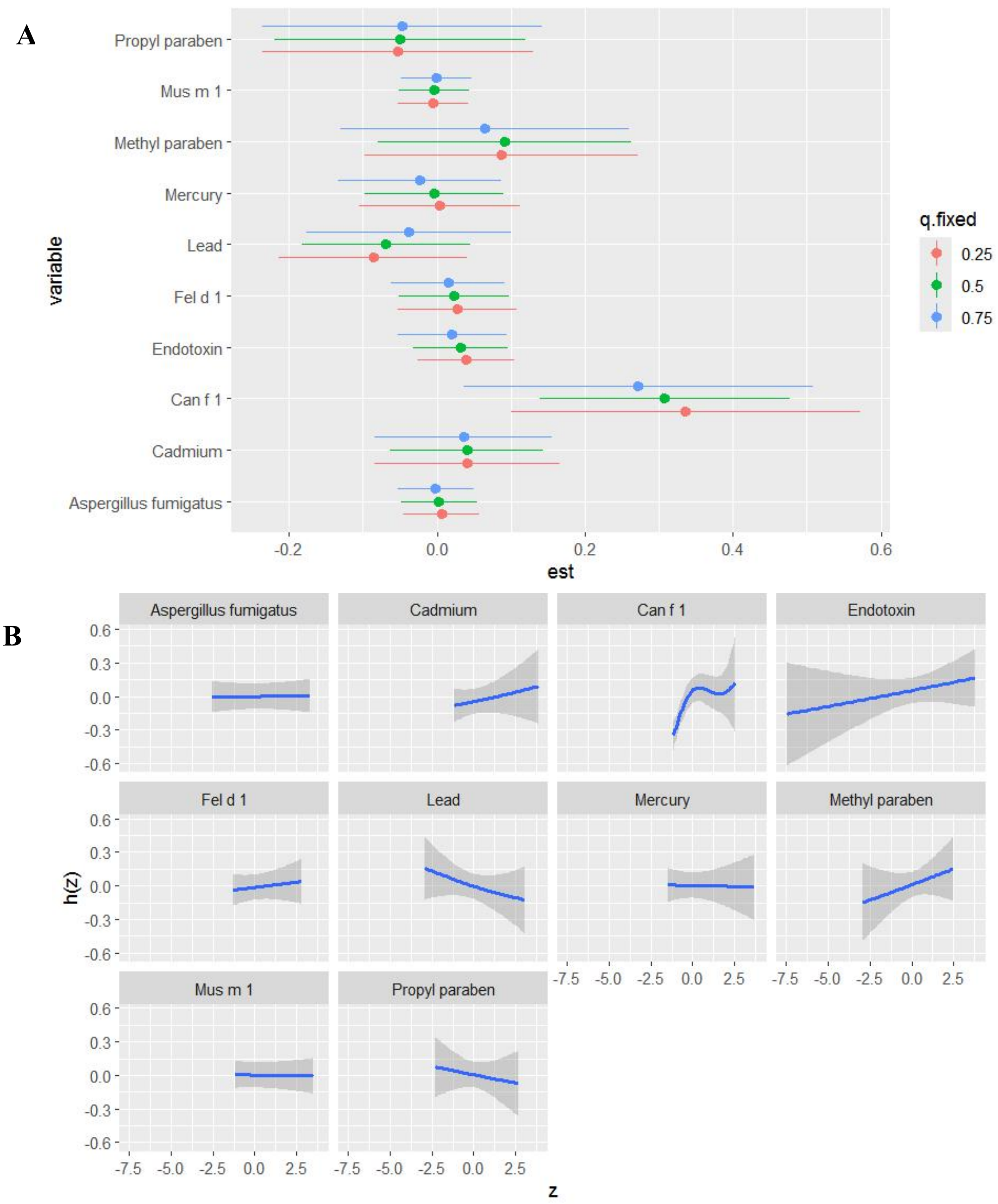

Supplementary Figure S3 (A)Associations of single exposure indicators with allergy-related outcomes risk were estimated by BKMR models in total population and subgroups, when other exposure indicators were held at their corresponding 25th (red), 50th (green) or 75th (blue) percentile, respectively. (B)Univariate exposure–response function (95% CI) between selected exposure indicator concentrations and allergy-related outcomes while fixing the concentrations of other exposure indicators at median values. The results were assessed by the BKMR model adjusted for sex, age, BMI, race, education level, annual household income, alcohol and serum cotinine, and ln-transformed creatinine.

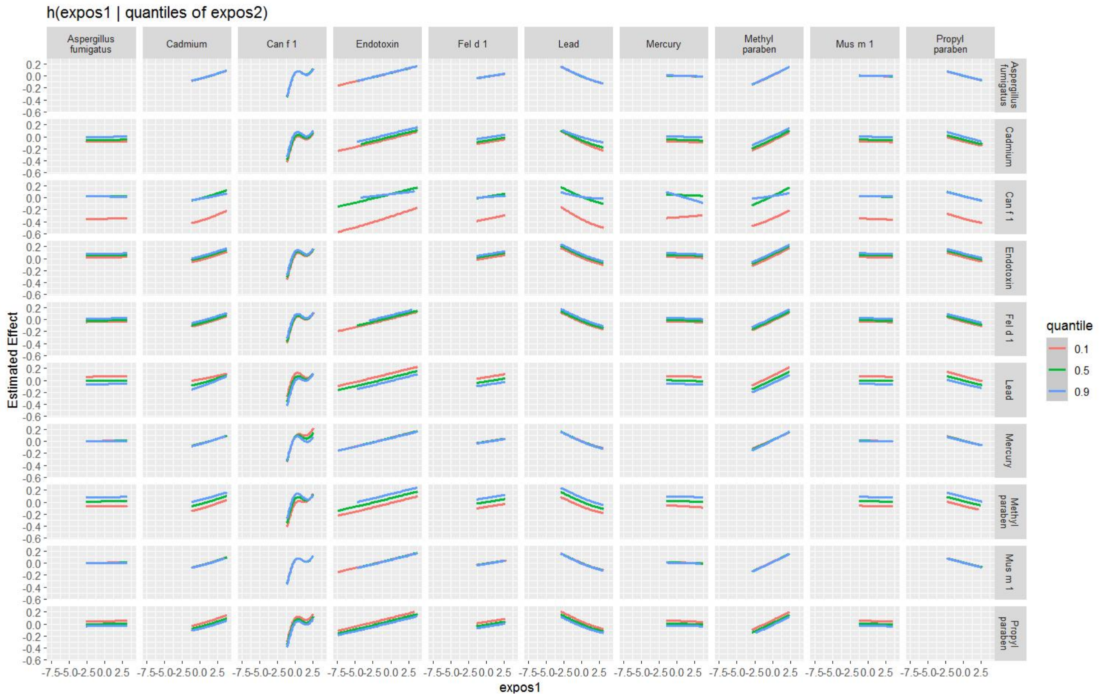

Supplementary Figure S4 Association between single exposure indicator with allergy-related outcomes, while fixing exposure 2 at the 10th, 50th, and 90th quantiles (and holding the remnant predictors to their median level). The models were adjusted for sex, age, BMI, race, education level, annual household income, alcohol and serum cotinine, and log-transformed creatinine.
